# The miR-27a-3p/FTO axis modifies hypoxia-induced malignant behaviors of glioma cells: miR-27a-3p/FTO affects glioma growth

**DOI:** 10.3724/abbs.2023002

**Published:** 2023-01-31

**Authors:** Peng Du, Li Meng, Xinbin Liao, Yi Liu, Xin Mo, Mengqi Gong, Yiwei Liao

**Affiliations:** 1 National Clinical Research Center for Geriatric Disorders Xiangya Hospital Central South University Changsha 410008 China; 2 Department of Neurosurgery Xiangya Hospital Central South University Changsha 410008 China; 3 Department of Neurosurgery The Second Affiliated Hospital Xinjiang Medical University Urumqi 830063 China; 4 Department of Radiology Xiangya Hospital Central South University Changsha 410008 China

**Keywords:** glioblastoma, m6A RNA methylation, FTO, miR-27a-3p, hypoxia

## Abstract

Glioblastoma multiforme (GBM) is one of the most malignant types of central nervous system (CNS) tumors. N6-methyladenine (m6A) RNA modification is a main type of RNA modification in eukaryotic cells. In this study, we find that the m6A RNA methylation eraser FTO is dramatically downregulated in glioma samples and cell lines, particularly in intermediate and core regions and hypoxia-challenged glioma cells. *In vitro*, FTO overexpression inhibits the hypoxia-induced capacities of glioma cells to proliferate, migrate and invade, and decreases the percentage of cells with m6A RNA methylation. *In vivo*, FTO overexpression inhibits tumor growth in the xenograft model and decreases the protein levels of migration markers, including Vimentin and Twist. miR-27a-3p is upregulated within glioma intermediate and core regions and hypoxia-challenged glioma cells. miR-27a-3p inhibits the expression of FTO via direct binding to FTO. miR-27a-3p overexpression promotes hypoxia-challenged glioma cell aggressiveness, whereas FTO overexpression partially diminishes the oncogenic effects of miR-27a-3p overexpression. FTO overexpression promotes the nuclear translocation of FOXO3a and upregulates the expression levels of the FOXO3a downstream targets BIM, BNIP3, BCL-6, and PUMA, possibly by interacting with FOXO3a. Conclusively, FTO serves as a tumor suppressor in glioma by suppressing hypoxia-induced malignant behaviors of glioma cells, possibly by promoting the nuclear translocation of FOXO3a and upregulating FOXO3a downstream targets. miR-27a-3p is a major contributor to FTO downregulation in glioma under hypoxia.

## Introduction

Glioblastoma multiforme (GBM) is one of the most malignant types of central nervous system (CNS) tumors [ 1, 2] . GBM still plagues physicians, despite medical and technological advances in cancer therapies, and patients with GBM have a dismal prognosis [3]. The hypoxic microenvironment in tumors might be the major contributor to GBM’s high aggressiveness. GBM commonly shows dense proliferation, and cancer cell hyperproliferation frequently results in increased oxygen demand. The tumor microcirculation is disturbed and hypoxic when the oxygen demand of cancer cells exceeds the oxygen supply provided by microvessels [4].

Non-coding RNAs and post-transcriptional alteration of RNAs have recently emerged as important topics of cancer research. Among them, N6-methyladenine (m6A) RNA modification is a key focus of study [ 5, 6] . m6A is installed cotranscriptionally through the methyltransferase complex (MTC), which consists of the methyltransferase-like 3 (METTL3) catalytic subunit and other accessory subunits, including METTL14 and Wilms tumor 1-associated protein (WTAP) [ 7– 9] . Two well-known eraser enzymes, alkylation repair homolog protein 5 (ALKBH5) and fat mass and obesity-associated protein (FTO), are involved in m6A removal [ 10, 11] . The YT521-B homology (YTH) domain family of proteins (YTHDF1, YTHDF2, YTHDF3, YTHDC1, and YTHDC2) are direct ‘readers’ of m6A that influence target mRNA translation, stability, and splicing [12]. m6A modification can affect tissue development, stem cell self-renewal and differentiation, the heat shock response, biological clock regulation, the DNA damage response, and the maternal-to-zygotic transition [ 8, 12] . m6A is a critical epitranscriptomic mark that is abundant in the CNS and exerts an important effect on brain development and function [13]. In addition, dysregulation of m6A modification has been linked to the development of different malignancies, including gliomas [6]. For example, TRMT61A is a downstream target of HIF1A and is downregulated after c-Myc suppression within GBM under hypoxic conditions [14]. Moreover, Xie *et al*. [15] revealed that hypoxia-associated gene profiles were the most enriched after ALKBH1 knockdown, and ALKBH1 regulated hypoxia response genes even when cells were cultured under indoor air conditions. Therefore, more m6A regulators might be involved in glioma tumorigenesis.

MicroRNAs (miRNAs), a class of small noncoding RNAs of approximately 22 nucleotides, can control a variety of biological processes by leading to mRNA degradation or translation inhibition [16]. A variety of miRNAs, which are changed by hypoxia, could target key oncogenes and tumor suppressors, therefore influencing tumorigenic processes [ 17, 18] . Initial studies of miRNA expression alterations in glioblastoma occurred in 2005 [ 19, 20] . These studies used microarray technology and Northern blotting to detect deregulated miRNAs in GBM cell lines and tumors, demonstrating overexpression of miR-21 [20] and miR-221 [19], as well as downregulation of miR-128 and miR-181a, miR-181b, and miR-181c [5]. Other high-throughput methods were later used to uncover other microRNA expression alterations in gliomas. Thus, miRNAs might be the bridge mediating glioma adaptation to hypoxia stress by modulating m6A RNA methylation regulators.

Herein, differentially expressed m6A RNA methylation regulators under normoxia/hypoxia were first analyzed and FTO was selected. The *in vitro* and *in vivo* effects of FTO on glioma cell responses to hypoxia stress and tumor growth in a xenograft model were investigated. miRNAs that were differentially expressed in hypoxia-challenged glioma cells and might target FTO were analyzed and miR-27a-3p was selected. Predicted miR-27a-3p binding and regulation of FTO were verified, and the dynamic effects of the miR-27a-3p/FTO axis on hypoxia-challenged glioma cells were investigated.

## Materials and Methods

### Clinical sampling

Fifteen glioma tissue samples from the tumor core region (tumor samples, core), fifteen glioma tissue samples from the tumor intermediate region (tumor samples, intermediate), and fifteen tissue samples from the peritumoral brain edema (PTBE; control normal samples) region were collected from patients undergoing surgical resection at Xiangya Hospital of Central South University (Changsha, China). Sampling was performed under the approval of Xiangya Hospital’s Ethics Committee (202009498). Informed consent was signed by each enrolled patient. Specimens were collected and promptly kept at –80°C until further usage.

### Cell lineages and culture

The U-251MG cell line (GDC0093) was purchased from the China Center for Type Culture Collection (CCTCC, Wuhan, China) and grown in MEM (Gibco, Waltham, USA) containing 10% FBS (Invitrogen, Waltham, USA). The LN229 cell line (CRL-2611) was obtained from ATCC (Manassas, USA) and grown in Dulbecco’s modified Eagle’s medium (DMEM, 30-2002; ATCC) containing 10% FBS (Invitrogen). For hypoxia treatment, cells were cultured in a sealed Modular Incubator Chamber (Billups-Rothenberg, Del Mar, USA) flushed with 1% O _2_, 5% CO _2_, and 94% N _2_, and incubated at 37°C for 24 h.

### Measurement of total m6A level

Total RNA was extracted from target tissue samples or cell lines using the RNeasy Mini kit (Qiagen, Hilden, Germany). The EpiQuik M6A RNA Methylation Quantification kit (EpiGentek Group, Inc., Farmingdale, USA) was employed to determine the m6A RNA methylation level following the manufacturer′s instructions.

### Western blot analysis

Western blot analysis was performed to determine the protein levels of HNRNPC, METTL14, METTL3, FTO, IGF2BP3, ALKBH5, IGF2BP2, PCNA, Vimentin, Twist, FOXO3a, BIM, BNIP3, BCL-6, and PUMA in target tissues and cell lines. For total protein isolation, RIPA lysis buffer was used to resuspend the target cells [21]. After 15 min on ice, the suspension was vortexed for 10 s before being centrifuged at 4°C for 10 min at 12,000 *g*. The supernatant was collected as the protein sample. For cytoplasmic and nuclear protein isolation, the Nuclear and Cytoplasmic Protein Extraction Kit (Beyotime, Shanghai, China) was used according to the manufacturer’s instructions. Next, a Bio-Rad protein detection kit (Bio-Rad, Hercules, USA) was employed to determine the protein sample concentration. After being loaded on SDS-PAGE gels for protein separation, the protein samples were transferred to PVDF membranes for detection using primary antibodies against HNRNPC, METTL14, METTL3, FTO, IGF2BP3, ALKBH5, IGF2BP2, PCNA, Vimentin, Twist, FOXO3a, BIM, BNIP3, BCL-6, and PUMA (Proteintech, Wuhan, China) at 4°C overnight, followed by incubation of the membranes with an HRP-conjugated secondary antibody (1:5000; Proteintech). The signals were observed using ECL substrates (Millipore, Billerica, USA). Tubulin was employed to normalize the protein expression as an internal reference for total protein and cytoplasmic protein. Histone H3 (H3) was employed as an internal reference for nuclear protein.

### qRT-PCR

Total cellular RNA, extracted from target cells or tissue samples by the use of TRIzol reagent (Invitrogen), was reverse transcribed successfully using the StarScript II First-strand cDNA synthesis kit (GenStar, Beijing, China) following the manufacturer’s protocols. SYBR Green PCR Master Mix (Takara, Dalian, China) was employed to determine the expression of target factors. The Ct method was employed to calculate the relative expression using *tubulin* or *U6* expression as an internal reference. The primers used are listed in Supplementary Table S1.

### Histopathological examinations

Histological investigations were carried out following previously established hematoxylin and eosin (H&E) and immunohistochemistry (IHC) staining procedures [22]. The clinical specimens were fixed overnight in 4% paraformaldehyde before being embedded in paraffin and sectioned at a thickness of 5 μm. The primary antibody against FTO was utilized for IHC as previously described [23]. Briefly, after blocking endogenous peroxidase and non-specific antibody binding sites, the sections were incubated with FTO antibody (1:200) at room temperature for 2 h and then further incubated with goat anti-rabbit/mouse HRP IgG (Proteintech) at room temperature for 30 min. Fresh prepared diaminobenzidine (DAB) substrate (Proteintech) was used as chromogen. After 5–10 min of incubation, the sections were re-stained with hematoxlyin for 2 min and observed under an optical microscope (Olympus, Tokyo, Japan).

### Cell transfection and infection

Target cells at a density of 1×10 ^6^ cells/well were seeded into 6-well plates and cultured for 24 h in growth medium with 10% FBS. Next, cells were transduced with lentivirus (lv) lv-sh-NC, lv-sh1-FTO, or lv-sh2-FTO (for FTO knockdown), lv-NC, lv-FTO (for FTO overexpression), or agomir-27a-3p/antagomir-27a-3p (for miR-27a-3p overexpression or inhibition) using polybrene (Beyotime) or Lipofectamine® 3000 reagent (Thermo Fisher Scientific, Waltham, USA) according to the manufacturer’s protocols. The sequences are shown in Supplementary Table S1.

### CCK-8

The Cell Counting Kit-8 assay (CCK-8) was used to measure target cell proliferation. Cells (2×10 ^3^ cells per well) were transfected and plated in 96-well plates. After 48 h, 10 μL of CCK-8 solution (Beyotime) was added to each well of the plate, followed by incubation for 2 h at 37°C. The absorbance at 450 nm was measured with a microplate reader (Molecular Devices, San Jose, USA).

### Wound healing assay

The migratory capacity of target cells was investigated by the wound healing assay. The initial seeding density of the tested cells was 2×10 ^5^ cells/cm ^2^. A scratch wound was made using a 100-μl pipette tip. The cells were rinsed twice in PBS before being cultured in serum-free culture medium at 37°C for 24 h. An inverted light microscope was used to examine the cells. The amount of wound healing was calculated using ImageJ software (National Institutes of Health, Bethesda, USA).

### Transwell assay

BD Biosciences′ Matrigel was thawed overnight at 4°C and diluted with DMEM. Sixty microliters of diluted Matrigel was added to the upper chambers of a 24-well Transwell insert, followed by incubation for 30 min at 37°C. Target cells at a density of 2×10 ^4^ cells per well in serum-free medium were seeded in the top chamber. In the lower compartment, 600 μl of culture medium supplemented with 10% FBS was introduced, followed by incubation at 37°C for 24 h. The noninvasive cells were removed from the top of the Transwell insert using a cotton swab. Methanol and 0.3% crystal violet stains were used to determine the rate of cell invasion.

### Xenograft tumor model in nude mice

Four-week-old Balb/c nude mice were acquired from the SLAC Experimental Animal Center (Changsha, China). The procedures were approved by the Animal Research Ethics Committee of the Xiangya Hospital of Central South University (202009499). The procedures involving animals and their care were in accordance with the ethical approval of Xiangya Hospital of Central South University. GBM cells were infected with lentivirus overexpressing FTO (Lv-FTO) or control lentivirus (Lv-NC), followed by subcutaneous injection with 2.5×10 ^5^ cells in a total volume of 200 μl into the right dorsal-lateral flanks of mice. Mice were sacrificed under anesthesia after 4 weeks, and the following formula was employed to estimate the tumor volume: tumor volume (mm ^3^)=length×width ^2^/2. Then, formalin-fixed paraffin-embedded tissue samples were prepared according to conventional histological examination protocols.

### Dual-luciferase reporter assay

By cloning the FTO 3′UTR into the psiCheck-2 vector (Promega, Madison, USA), a wild-type FTO 3′UTR reporter vector was created. Mutating the anticipated miR-27a-3p binding location in the FTO 3′UTR resulted in mutant-type vectors. The reporter vectors were then co-transduced with agomir-27a-3p/antagomir-27a-3p into tool cells (293T). The luciferase activity was measured as previously described [ 23, 24] .

### Statistical analysis

All statistical analyses were performed with SPSS v20 (SPSS Inc., Chicago, USA), and the results are reported as the mean±SD of at least three independent experiments. To examine the differences between the two groups, an unpaired *t*-test was employed. One-way ANOVA with Tukey’s post hoc test was used to compare various groups. Then, if all data met the assumption of homogeneity of variances, Tukey’s post hoc test was used.

## Results

### The expression of m6A RNA methylation regulators is altered in glioma tissues and cells

**Figure 1:**
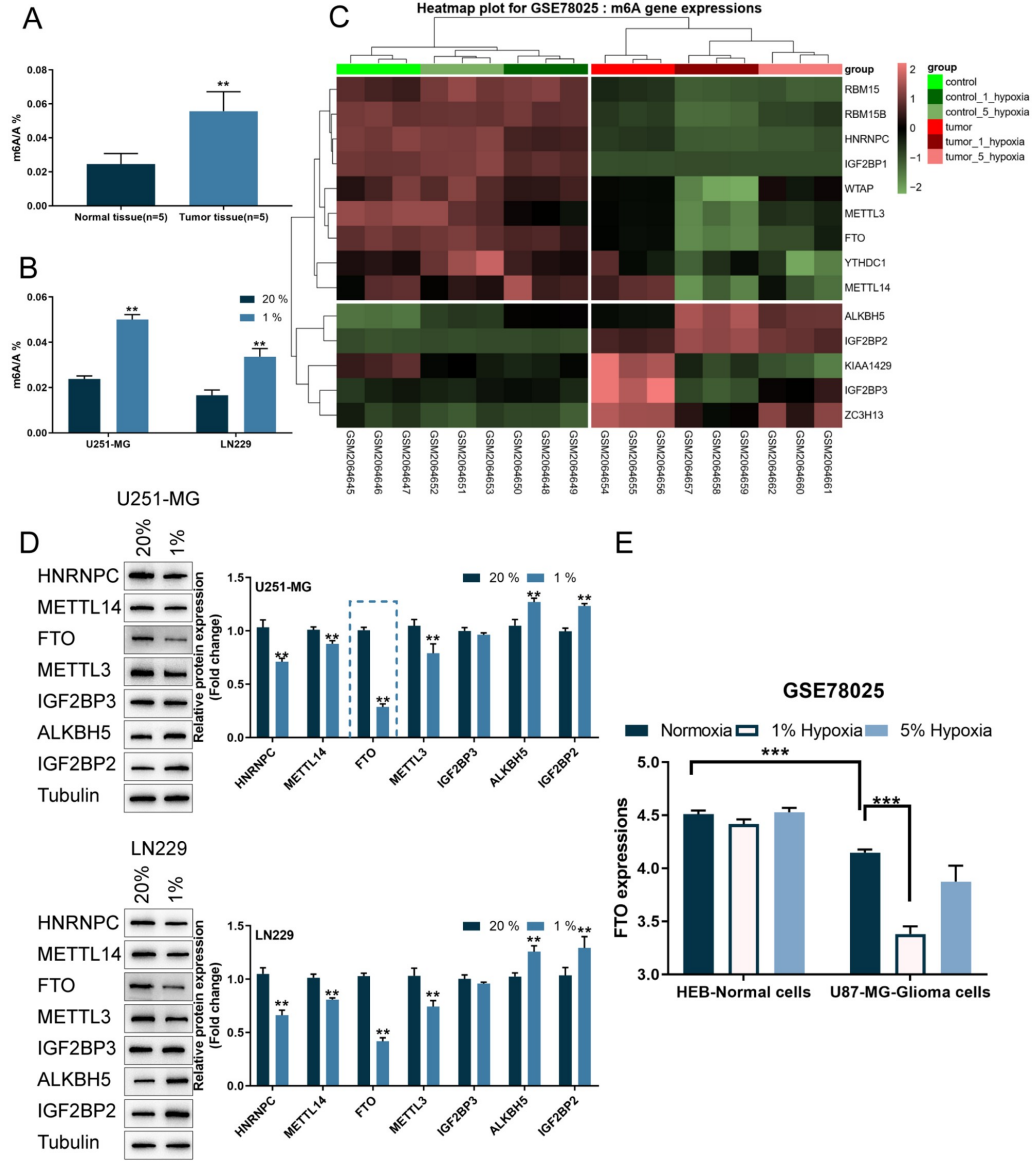
Figure 1 The expression of m6A RNA methylation regulators is altered in glioma tissues and cells (A,B) m6A RNA methylation levels in glioma tissues and cell lines determined using an EpiQuik M6A RNA Methylation Quantification kit. (C) Top differentially expressed m6A methylation regulators in the U87-MG cell line as a human GBM cell model and the human brain HEB cell line as a non-neoplastic brain cell line under normoxia or hypoxia, according to GSE78025. (D) U251-MG and LN229 cells were exposed to 1% or 20% oxygen (hypoxia or normoxia) and the protein levels of HNRNPC, METTL14, FTO, METTL3, IGF2BP3, ALKBH5, and IGF2BP2 were determined by western blot analysis. (E) According to GSE78025, FTO expression was downregulated in glioma cells challenged by hypoxia compared with glioma cells under normoxia. ** P<0.01, *** P<0.001.

We employed an EpiQuik M6A RNA Methylation Quantification kit to first assess the levels of m6A RNA methylation within collected glioma tissues and cell lines exposed to hypoxia or normoxia. Figure 1A shows that the m6A RNA methylation level was significantly higher within glioma tissue samples than in normal tissues (peritumoral brain edema). Furthermore, the level of m6A RNA methylation was greater in glioma cell lines exposed to hypoxia (1% oxygen) than in those exposed to normoxia ( Figure 1B). To identify the predominant m6A RNA methylation regulators in hypoxia-challenged glioma cells, differentially expressed regulators were analyzed in the U87-MG cell line (GBM cell line) and HEB (normal brain glial cell line) under normoxia or hypoxia, according to GSE78025 and GSE77307. Figure 1C and Supplementary Figure S1A,B show the top differentially expressed regulators, according to GSE78025 and GSE77307. Table 1 shows 7 overlapping regulators (5 upregulated and 2 downregulated) in both datasets. We exposed U251-MG and LN229 cells to 1% (hypoxia) or 20% (normoxia) oxygen and determined the protein levels of the regulators mentioned above, including HNRNPC, METTL14, FTO, METTL3, IGF2BP3, ALKBH5, and IGF2BP2. Among all tested regulators, FTO showed the greatest change in protein level ( Figure 1D). Moreover, according to GSE78025 and GSE77307, FTO expression was downregulated in glioma samples compared with that in normal healthy samples and downregulated in glioma cells challenged by hypoxia compared with glioma cells under normoxia ( Figure 1E and Supplementary Figure S1C). Therefore, FTO was selected for further experiments.

**Table:** **Table 1** Differentially expressed m6A RNA methylation regulators in glioma

Gene	logFC	AveExpr	*t*	*P* Value	adj. *P*.Val	B	Change
*ALKBH5*	0.825239	6.323291	27.538130	3.05E-06	4.64E-05	5.127886	UP
*IGF2BP2*	0.730759	4.531312	26.037320	3.94E-06	5.44E-05	4.825264	UP
*YTHDC1*	0.295450	3.565253	5.524470	0.003554	0.009267	–3.364500	NOT
*ZC3H13*	0.166128	3.582529	6.370006	0.001979	0.005659	–2.665110	NOT
*KIAA1429*	0.076765	4.154540	2.725677	0.045799	0.085858	–6.345260	NOT
*IGF2BP1*	0.076153	0.067044	2.043151	0.101989	0.172262	–7.225170	NOT
*RBM15*	–0.060640	2.727765	–1.464200	0.208528	0.321233	–7.960380	NOT
*RBM15B*	–0.093810	3.968426	–2.495490	0.059557	0.108520	–6.638680	NOT
*WTAP*	–0.273380	5.062251	–5.164530	0.004659	0.011720	–3.686880	NOT
*HNRNPC*	–0.328170	6.454020	–13.339800	7.99E-05	0.000444	1.203929	DOWN
*METTL14*	–0.339310	2.410188	–4.436300	0.008441	0.019540	–4.390300	DOWN
*FTO*	–0.397150	3.856680	–9.953280	0.000292	0.001208	–0.361510	DOWN
*METTL3*	–0.422680	3.729892	–5.520980	0.003563	0.009289	–3.367550	DOWN
*IGF2BP3*	–0.718690	4.604235	–11.726900	0.000142	0.000683	0.511793	DOWN

### FTO expression is decreased in glioma and correlated with prognosis

**Figure 2:**
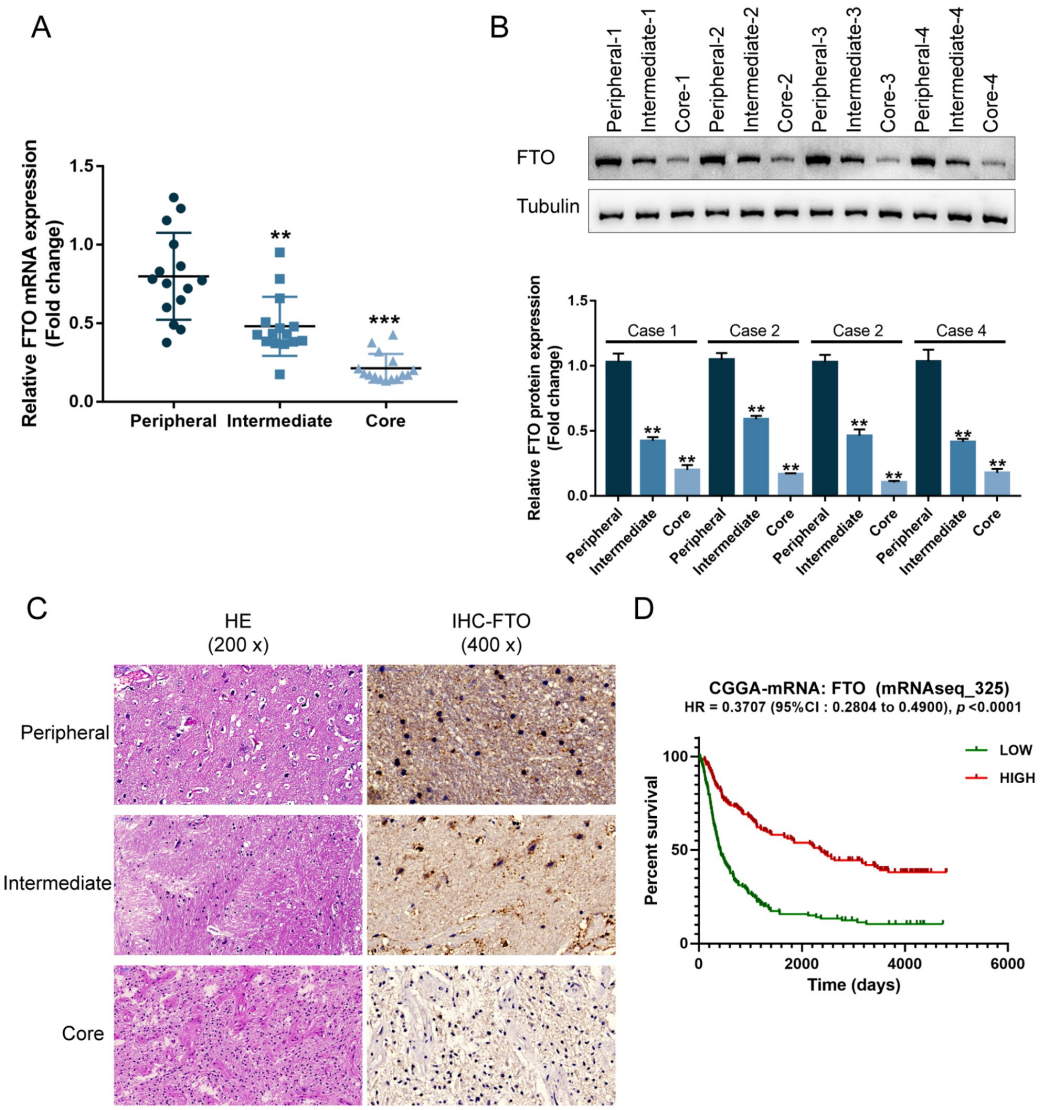
Figure 2 FTO expression is decreased in glioma and correlated with prognosis (A) FTO mRNA expression in the core, intermediate, and peripheral regions of glioma examined by qRT-PCR. (B) FTO protein levels in the core, intermediate, and peripheral regions of glioma were examined by western blot analysis. (C) FTO levels and distribution in the core, intermediate, and peripheral regions of glioma were examined by immunohistochemical (IHC) staining. (D) Cases from Chinese Glioma Genome Atlas (CGGA)-mRNAseq (n=325) were assigned into high- and low-FTO expression groups using the median expression value as the cut-off; the correlation between FTO expression and patient survival was analyzed by the R language package Survminer and Survival. ** P<0.01, *** P<0.001.

Before investigating the specific effects of FTO *in vitro* and *in vivo*, we first examined FTO expression in different regions of glioma. Figure 2A shows that, compared with the core region, the mRNA expression level of FTO was dramatically higher within intermediate and peripheral regions of glioma, especially in the peripheral region. Consistently, FTO protein level was lower in the core and intermediate regions, particularly within the core region, than in the peripheral region of glioma ( Figure 2B,C). We divided cases from the Chinese Glioma Genome Atlas (CGGA)-mRNAseq ( *n*=325) into high- and low-FTO expression groups based on the median FTO expression; the association between the expression level of FTO and the survival of glioma patients was analyzed using the R language package Survminer and Survival [ 25, 26] . Figure 2D shows that higher FTO expression was correlated with better prognosis in glioma patients.

### FTO suppresses glioma cells growth, invasion and migration under normoxia or hypoxia

**Figure 3:**
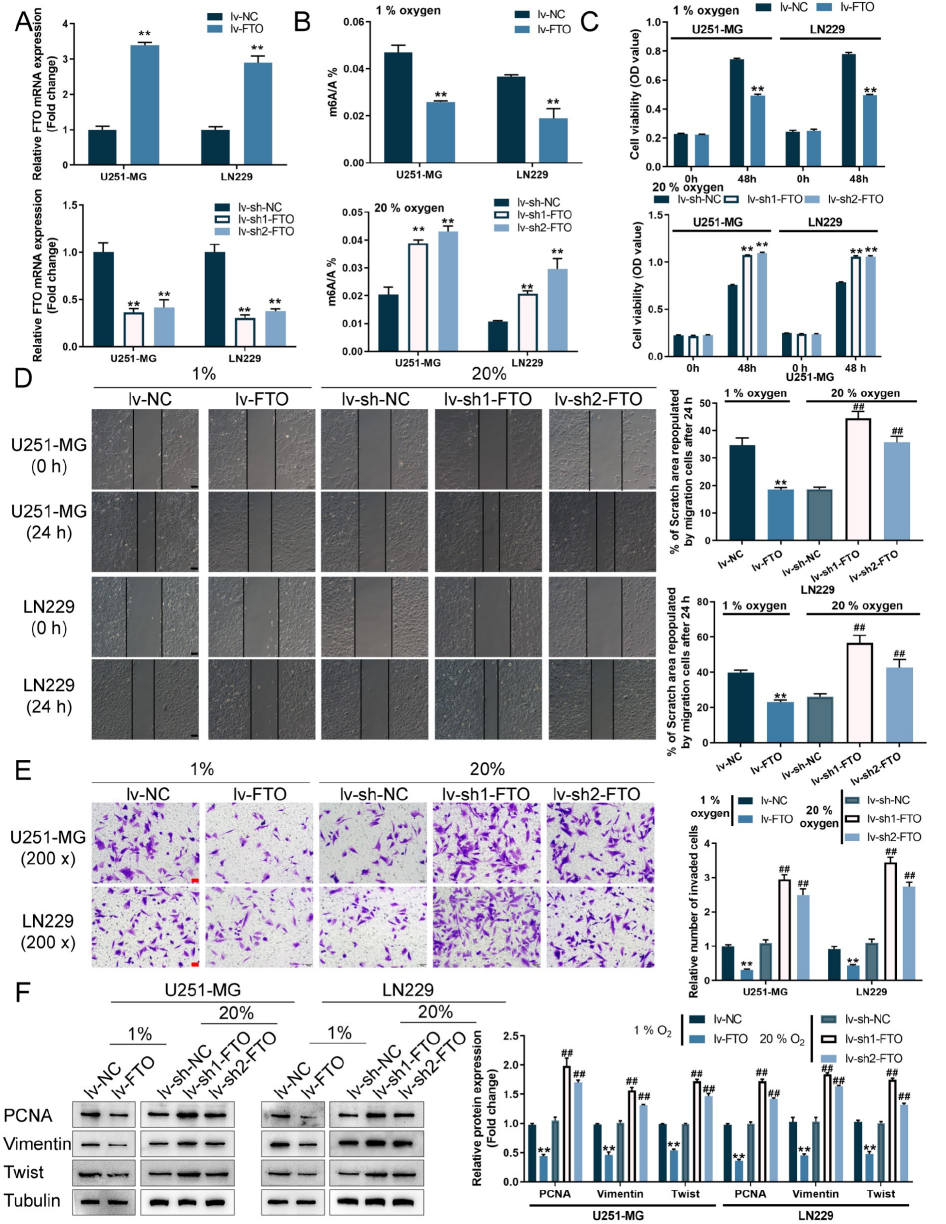
Figure 3 FTO suppresses glioma cells growth, invasion and migration under normoxia or hypoxia (A) FTO overexpression or knockdown was achieved in U251-MG and LN229 cells by transducing lentivirus overexpressing FTO (Lv-FTO) or lentivirus containing short hairpin RNA targeting FTO (Lv-sh1-FTO/Lv-sh2-FTO); transduction efficiency was confirmed by qRT-PCR. U251-MG and LN229 cells were subsequently transduced with lv-NV/lv-FTO and exposed to 1% oxygen (hypoxia) or transduced with lv-sh-NC/lv-sh1-FTO/lv-sh2-FTO and exposed to 20% oxygen (normoxia) and examined for m6A RNA methylation levels (B); cell viability using CCK-8 assay (C); cell migration using wound healing assay (D); cell invasion using Transwell assay (E); and the protein levels of PCNA, vimentin, and twist measured by western blot analysis (F). ** P<0.01 vs lv-NC group; ## P<0.01 vs lv-sh-NC group.

Given that the hypoxic microenvironment is a vital feature of the glioma core region, the *in vitro* effects of FTO on glioma cells were investigated under normoxia and hypoxia. We transduced lentivirus overexpressing FTO (Lv-FTO) or lentivirus containing short hairpin RNA targeting FTO (Lv-sh1-FTO/Lv-sh2-FTO) to achieve FTO overexpression or knockdown within U251-MG and LN229 cell lines, as confirmed by qRT-PCR ( Figure 3A). U251-MG and LN229 cells were subsequently transduced with lv-NV/lv-FTO and exposed to 1% oxygen (hypoxia) or transduced with lv-sh-NC/lv-sh1-FTO/lv-sh2-FTO and exposed to 20% oxygen (normoxia); the changes in m6A RNA methylation after FTO overexpression or knockdown were determined. In FTO-overexpressing glioma cells exposed to hypoxia, the percentage of cells with m6A RNA methylation was significantly decreased; in contrast, under normoxia, FTO knockdown significantly increased the percentage of m6A RNA-methylated cells ( Figure 3B). Next, we determined cell phenotypes. Under hypoxia, FTO overexpression inhibited glioma cell viability ( Figure 3C), cell migration ( Figure 3D), and cell invasion ( Figure 3E). Under normoxia, FTO knockdown enhanced the viability ( Figure 3C), migration ( Figure 3D), and invasion ( Figure 3E) of glioma cells. Consistently, under hypoxia, FTO overexpression decreased the proliferation marker PCNA and the cell migration markers Vimentin and Twist; in contrast, under normoxia, FTO knockdown increased the protein levels of PCNA, Vimentin, and Twist ( Figure 3F). Thus, under hypoxia, FTO serves as a tumor suppressor in glioma cells.

### FTO suppresses xenograft tumor growth in nude mice

**Figure 4:**
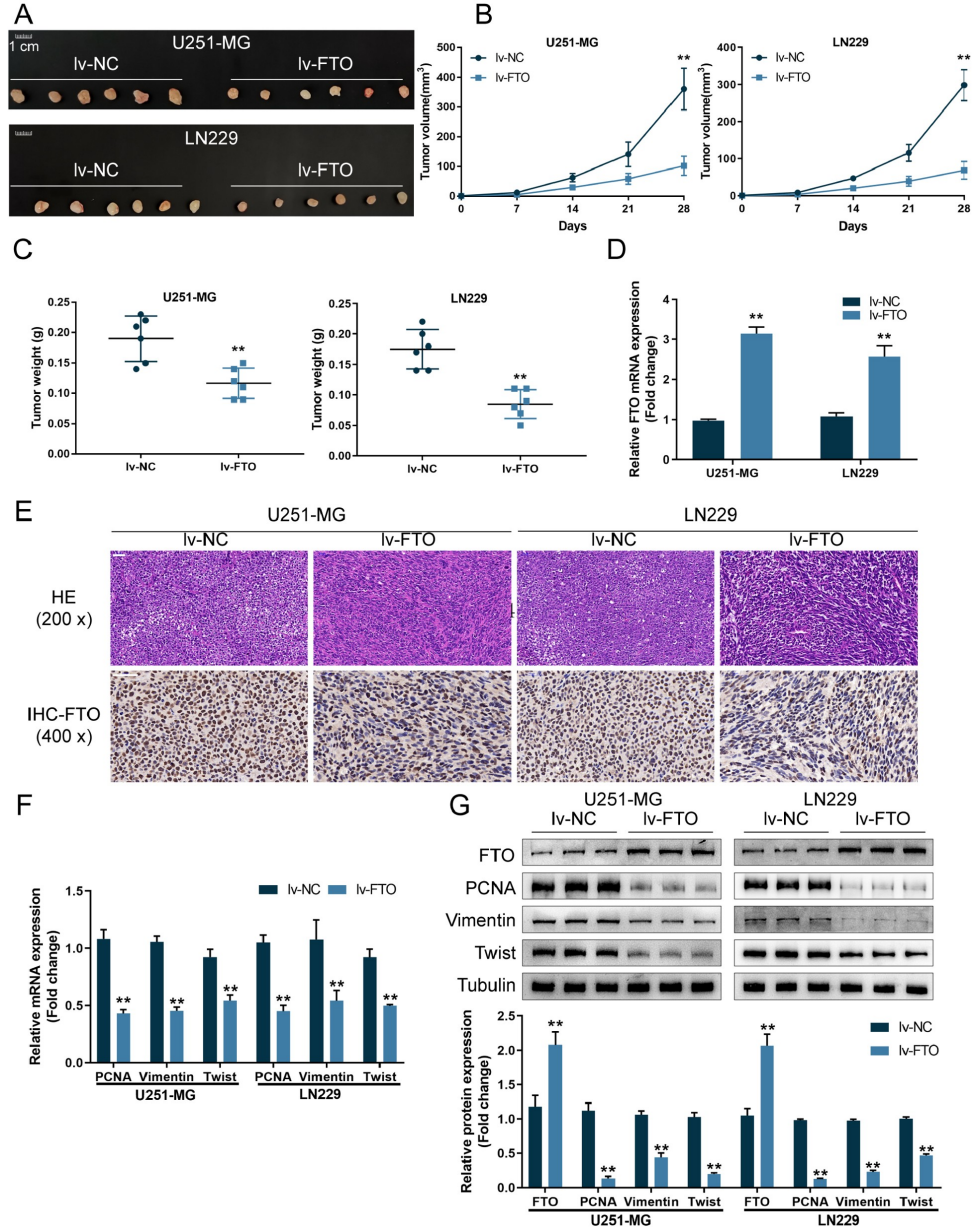
Figure 4 FTO inhibits xenograft tumor growth in nude mice (A) A xenograft tumor model was established in mice by injecting U251-MG or LN229 cells transduced with lv-NC/lv-FTO. (B) Tumor volumes were examined every 7 days until day 28 after injection. (C) Mice were sacrificed under anesthesia, and tumor weight was examined. (D) FTO mRNA levels in tumor tissues were examined by qRT-PCR. (E) Histopathological alterations and FTO levels in tumor tissues were evaluated by H&E and IHC staining. (F) PCNA, Vimentin and Twist mRNA levels in tumor tissues were examined by qRT-PCR. (G) The protein levels of FTO, PCNA, Vimentin, and Twist in tumor tissues were examined by western blot analysis. ** P<0.01.

To investigate the *in vivo* effects of FTO, we generated a xenograft tumor model in mice via injection of U251-MG/LN229 cell lines transduced with lv-NC/lv-FTO ( Figure 4A). Figure 4B shows that FTO overexpression reduced the tumor volumes during tumor growth in the xenograft model. On day 28, mice were sacrificed under anesthesia, and tumor weight was examined; FTO overexpression significantly decreased the tumor weight ( Figure 4C). The mRNA content of FTO was significantly increased in tumor tissue samples compared with the lv-NC group ( Figure 4D). Histopathological examination also indicated higher FTO levels in tumor tissues ( Figure 4E). qRT-PCR demonstrated that the mRNA expression of PCNA, Vimentin and Twist was downregulated in tumor tissue samples compared with that in the lv-NC group ( Figure 4F). Consistently, in tissue samples from the lv-FTO group, FTO protein levels were increased, whereas PCNA, Vimentin, and Twist protein levels were reduced ( Figure 4G).

### miR-27a-3p inhibits FTO expression in glioma cells

**Figure 5:**
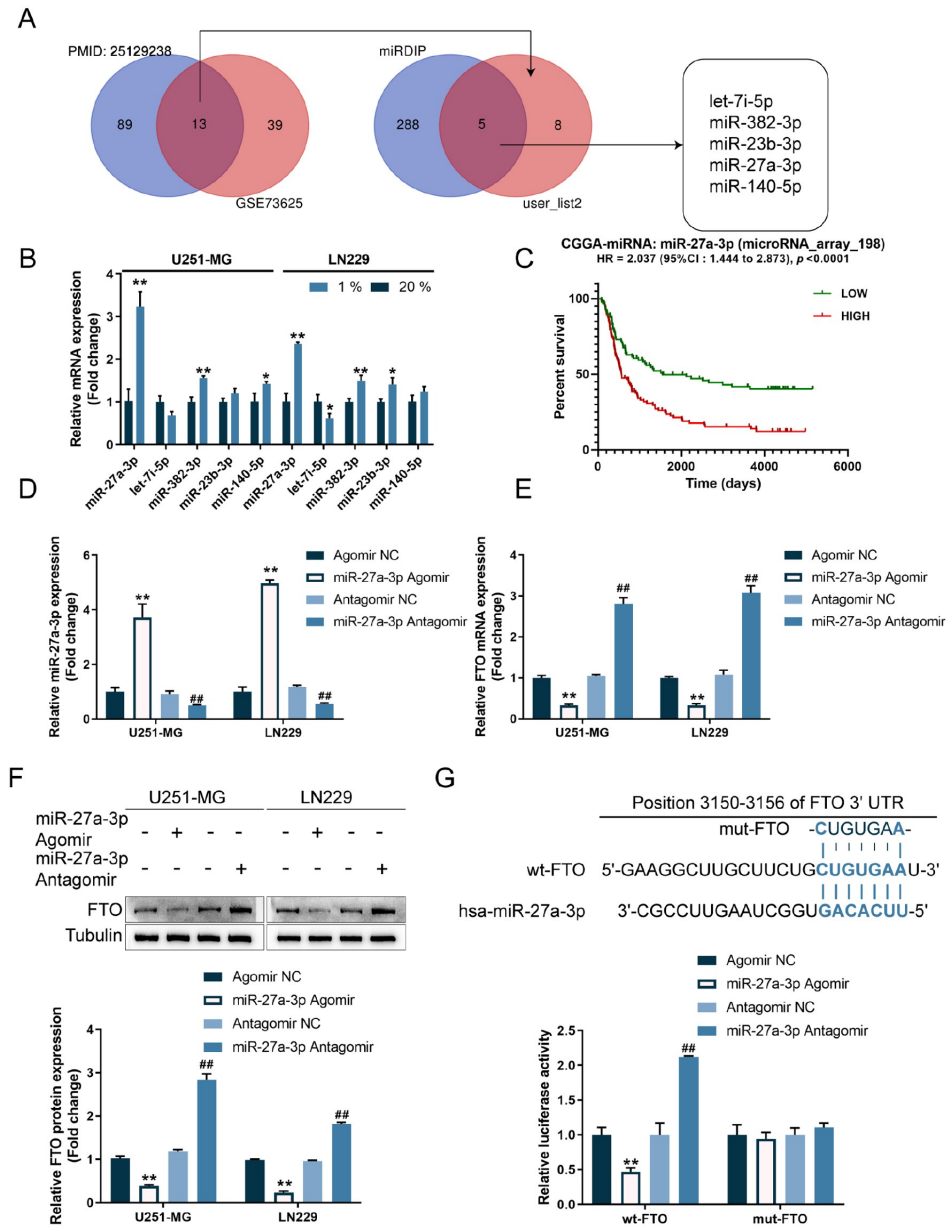
Figure 5 miR-27a-3p inhibits FTO expression in glioma cells (A) A schematic diagram of selecting miRNAs that might target FTO to inhibit FTO. (B) U251-MG and LN229 cells were exposed to 1% or 20% oxygen (hypoxia or normoxia) and examined for the expressions of let-7i-5p, miR-382-3p, miR-23b-3p, miR-27a-3p, and miR-140-5p by qRT-PCR. (C) Cases from CGGA-miRNAseq ( n=198) were assigned into high- and low-miR-27a-3p expression groups using the median expression value as the cut-off; the correlation between miR-27a-3p expression and patient survival was analyzed using the R language package Survminer and Survival. (D) miR-27a-3p overexpression or inhibition was achieved in U251-MG and LN229 cells by transducing with agomir-27a-3p or antagomir-27a-3p; transduction efficiency was confirmed by qRT-PCR. (E,F) U251-MG and LN229 cells were transduced with agomir-27a-3p or antagomir-27a-3p and examined for FTO mRNA expression by qRT-PCR and FTO protein level by western blot analysis. (G) A dual-luciferase reporter assay was performed to examine the luciferase activity in cells cotransduced with wild-type or mutant FTO reporter plasmids and agomir-27a-3p or antagomir-27a-3p. * P<0.05, ** P<0.01 vs 20% oxygen group or Agomir NC group; ## P<0.01 vs Antagomir NC group.

Given that miRNAs can target mRNAs, leading to mRNA degradation or translation inhibition [27], miRNAs that might target FTO to inhibit FTO were analyzed. GSE73625 used RNA sequencing to detail the global change in miRNA expression in glioma-initiating cells cultured under hypoxia compared to normoxia and identified deregulated miRNAs during hypoxia. Agrawal *et al*. [28] identified a set of hypoxia-upregulated miRNAs based on deep sequencing and microarray data (PMID:25129238). We compared these two datasets, and the intersection included let-7i-5p, miR-139-5p, miR-132-3p, miR-497-5p, miR-432-5p, miR-342-3p, miR-193b-3p, let-7i-3p, miR-23b-3p, miR-34c-5p, miR-27a-3p, miR-382-3p, and miR-140-5p. Among these miRNAs, let-7i-5p, miR-382-3p, miR-23b-3p, miR-27a-3p, and miR-140-5p were predicted by miRDIP to target FTO ( Figure 5A). We exposed U251-MG and LN229 cell lines to 1% (hypoxia) or 20% (normoxia) oxygen and determined let-7i-5p, miR-382-3p, miR-23b-3p, miR-27a-3p, and miR-140-5p expression using qRT-PCR; Figure 5B shows a sharp upregulation of miR-27a-3p in hypoxia-challenged glioma cells. Cases from CGGA-miRNAseq ( *n*=198) were assigned to high- and low-miR-27a-3p expression groups based on the median expression value; the association between the expression level of miR-27a-3p and patient survival was analyzed by the R language package Survminer and Survival. As inferred from Figure 5C, unlike FTO, higher expression of miR-27a-3p predicted poorer prognosis in glioma patients. Thus, we selected miR-27a-3p for further experiments.

Next, we transduced agomir-27a-3p/antagomir-27a-3p to achieve miR-27a-3p overexpression/inhibition within U251-MG and LN229 cell lines, as confirmed by qRT-PCR ( Figure 5D). In U251-MG and LN229 cells, miR-27a-3p overexpression downregulated FTO mRNA expression and decreased FTO protein levels, whereas miR-27a-3p inhibition exerted the opposite effects on FTO levels ( Figure 5E,F). To confirm the predicted miR-27a-3p binding to the FTO 3′UTR, we conducted a dual-luciferase reporter assay. In cells co-transduced with wt-FTO 3′UTR and agomir-27a-3p/antagomir-27a-3p, miR-27a-3p overexpression suppressed, while miR-27a-3p inhibition promoted, wt-FTO 3′UTR luciferase activity; in cells co-transduced with mut-FTO 3′UTR and agomir-27a-3p/antagomir-27a-3p, miR-27a-3p overexpression/inhibition caused no changes in luciferase activity ( Figure 5G). In summary, miR-27a-3p directly binds with the FTO 3′UTR and inhibits FTO expression.

### The miR-27a-3p/FTO axis modulates hypoxia-challenged glioma cells growth, migration and invasion

**Figure 6:**
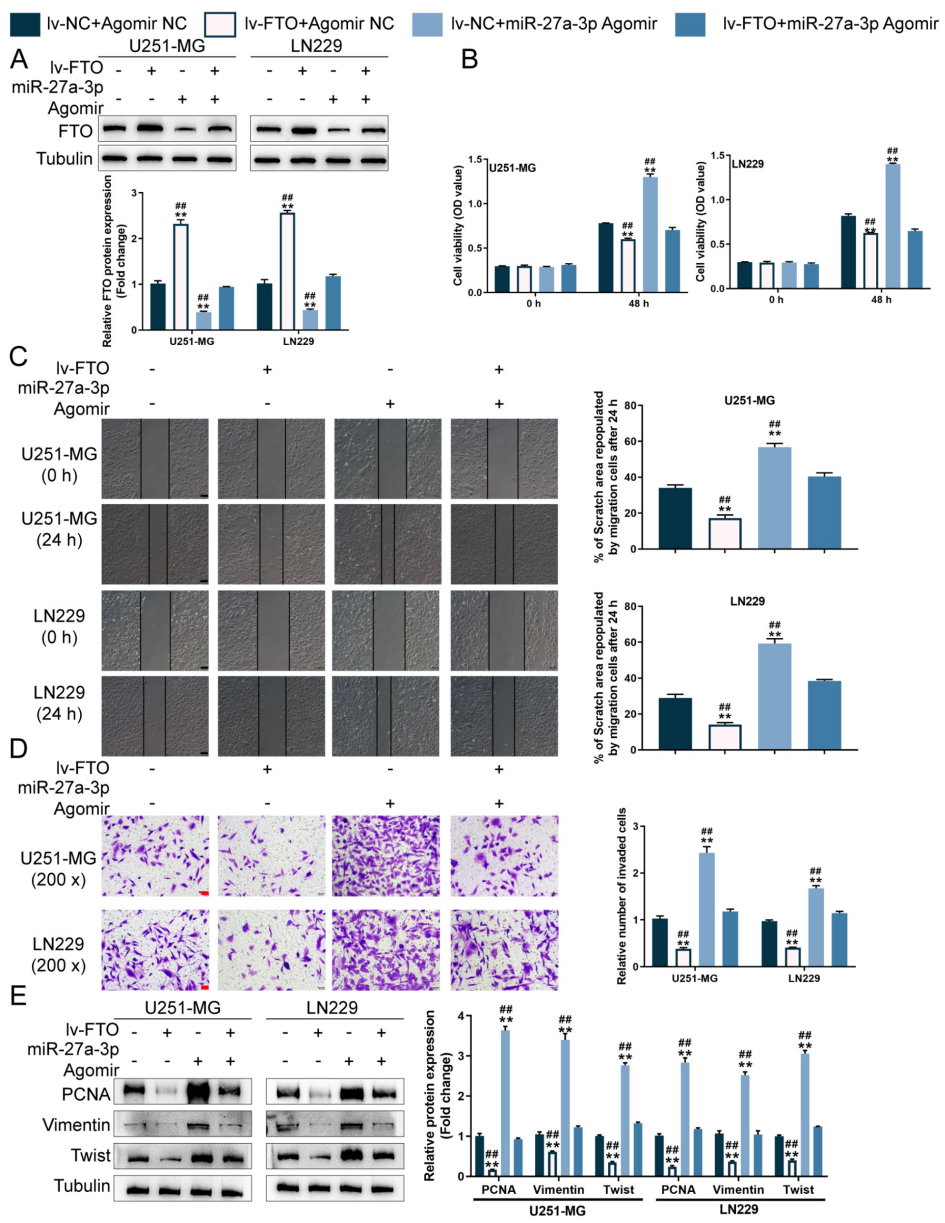
Figure 6 The miR-27a-3p/FTO axis modulates hypoxia-challenged glioma cells growth, migration and invasion U251-MG and LN229 cells were cotransduced with lv-FTO and agomir-27a-3p, exposed to 1% oxygen (hypoxia), and examined for FTO protein level by western blot analysis (A); cell viability using CCK-8 assay (B); cell migration using wound healing assay (C); cell invasion using Transwell assay (D); the protein levels of PCNA, vimentin, and twist by western blot analysis (E). ** P<0.01 vs lv-NC+Agomir NC group; ## P<0.01 vs lv-FTO+miR-27a-3p Agomir group.

After confirming the binding of miR-27a-3p to FTO, we investigated the dynamic effects of the miR-27a-3p/FTO axis. We co-transduced U251-MG and LN229 cell lines with lv-FTO and agomir-27a-3p, exposed these cells to 1% oxygen (hypoxia) and determined the protein contents of FTO. Figure 6A shows that under hypoxia, lv-FTO transduction increased, whereas agomir-27a-3p transduction decreased FTO protein level; the suppressive effects of agomir-27a-3p were partially diminished by lv-FTO. Consistently, under hypoxia, lv-FTO transduction suppressed glioma cell viability ( Figure 6B), cell migration ( Figure 6C), and cell invasion ( Figure 6D), whereas agomir-27a-3p exerted opposite effects on glioma cell phenotypes ( Figure 6B–D); similarly, the oncogenic effects of agomir-27a-3p on glioma cells were partially diminished by lv-FTO ( Figure 6B–D). Under hypoxia, lv-FTO transduction decreased the protein levels of PCNA, vimentin, and twist, whereas agomir-27a-3p increased these factors; the promotive effects of agomir-27a-3p on these factors were partially abolished by lv-FTO ( Figure 6E).

### FTO affects FOXO3a nuclear translocation and target gene expression

**Figure 7:**
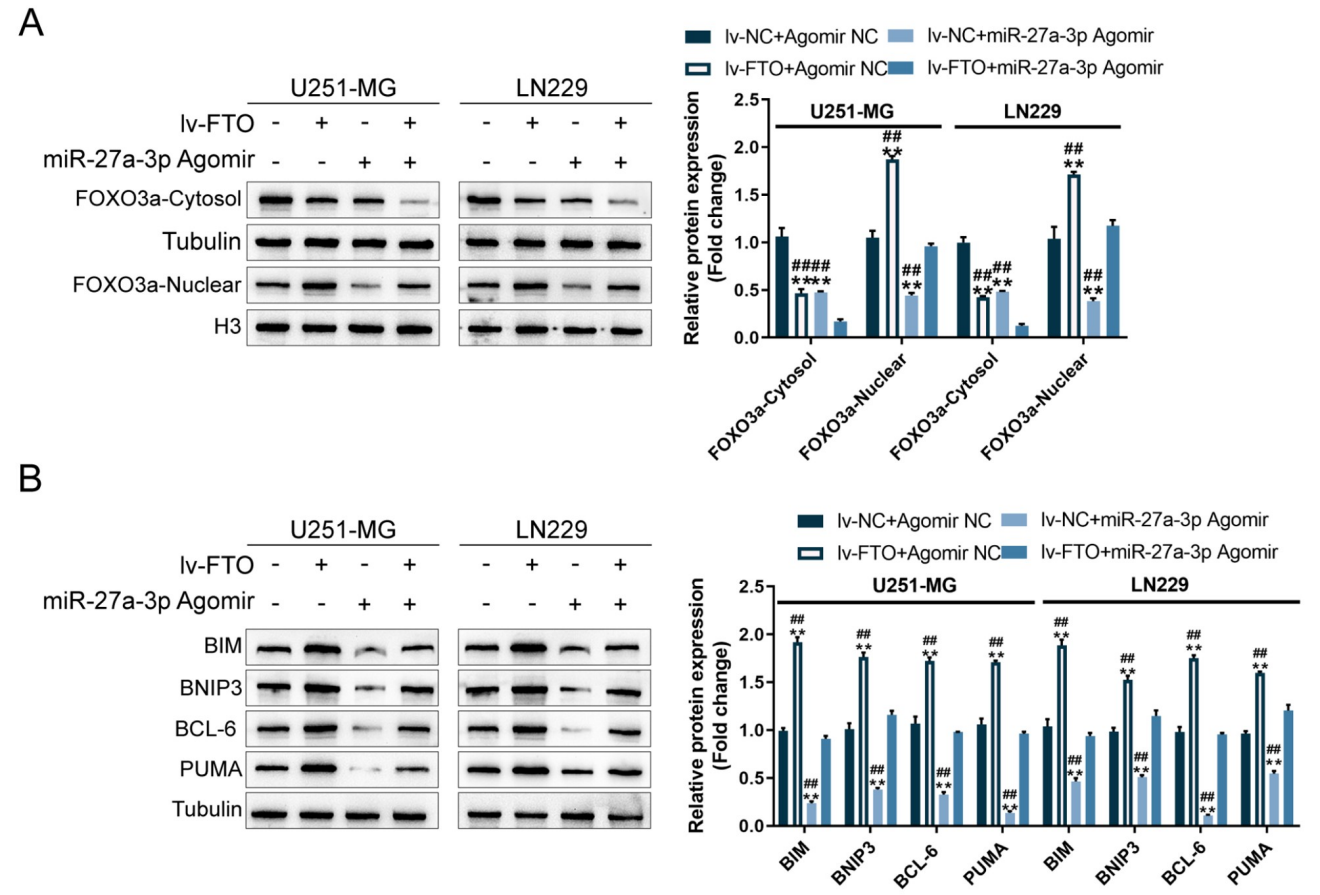
Figure 7 FTO affects FOXO3a nuclear translocation and target gene expression U251-MG and LN229 cells were co-transduced with lv-FTO and agomir-27a-3p and examined for the protein levels of nucleus-/cytoplasm-FOXO3a (A), BIM, BNIP3, BCL-6, and PUMA by western blot analysis (B). ** P<0.01 vs lv-NC+Agomir NC group; ## P<0.01 vs lv-FTO+miR-27a-3p Agomir group.

Since FTO has been reported to serve as a tumor suppressor in gliomas by interacting with FOXO3a, enhancing FOXO3a nuclear translocation and target gene expression [29], in this study, FTO regulation of FOXO3a nuclear translocation and the expression levels of reported FOXO3a targets (BIM, BNIP3, BCL-6, and PUMA) were investigated. U251-MG and LN229 cells were co-transduced with lv-FTO and agomir-27a-3p and examined for the protein levels of nuclear/cytoplasmic FOXO3a, BIM, BNIP3, BCL-6, and PUMA. Regarding FOXO3a nuclear translocation, FTO overexpression dramatically increased, whereas miR-27a-3p overexpression decreased, the level of nuclear FOXO3a; the suppressive effects of miR-27a-3p overexpression on FOXO3a nuclear translocation were partially diminished by FTO overexpression ( Figure 7A). Concerning FOXO3a targets, FTO overexpression significantly increased, whereas miR-27a-3p overexpression decreased, the levels of BIM, BNIP3, BCL-6, and PUMA; similarly, FTO overexpression partially eliminated the inhibitory effects of miR-27a-3p overexpression on FOXO3a targets ( Figure 7B).

## Discussion

In this study, we found that the m6A RNA methylation eraser FTO was dramatically down-regulated in glioma samples and cell lines, particularly in the intermediate and core regions and hypoxia-challenged glioma cells. *In vitro*, FTO overexpression inhibited the hypoxia-induced capacities of glioma cells to proliferate, migrate and invade and decreased the percentage of cells with m6A RNA methylation. *In vivo*, FTO overexpression inhibited tumor growth in the xenograft model and decreased the protein levels of migration markers, including Vimentin and Twist. miR-27a-3p was upregulated within glioma intermediate and core regions and hypoxia-challenged glioma cells. miR-27a-3p inhibited the expression of FTO via direct binding to FTO. miR-27a-3p overexpression promoted hypoxia-challenged glioma cell aggressiveness, whereas FTO overexpression partially diminished the oncogenic effects of miR-27a-3p overexpression. FTO overexpression promoted the nuclear translocation of FOXO3a and up-regulated the expression levels of the FOXO3a downstream targets BIM, BNIP3, BCL-6, and PUMA, possibly by interacting with FOXO3a. Although the roles of FTO and miR-27a in glioma have been widely recognized, their functions in a hypoxic microenvironment remain unclear. Considering the primary role of the hypoxic microenvironment in glioma chemo/drug resistance [ 30, 31] , this study first demonstrated the roles of miR-27a/FTO in glioma phenotypes, investigated the mechanisms from the aspects of m6A methylation regulation and the hypoxic microenvironment of glioma, which might provide new directions for clinical translation.

Methylation of RNA has recently been identified as a key carcinogenesis modifier. Many steps of mRNA metabolism are influenced by m6A methylation, the most common internal modification associated with eukaryotic mRNAs [32]. It is reasonable to believe that genetic variants in m6A regulators, such as writers, erasers, and readers, are involved in cancer development [33]. FTO has been found to act as an ‘eraser′ for m6A demethylation and has been linked not only to an increase in body mass and obesity [34] but also to oncogenesis [35]. Previous research has evaluated the link between FTO SNPs (single nucleotide polymorphisms) and carcinogenesis within prostate, pancreatic, breast and colorectal cancer [ 35, 36] . Nevertheless, the involvement of FTO as an m6A demethylase in cancer progression has only recently been discovered. As previously reported, the expression of FTO is elevated within tumors, which enhances gastric cancer proliferation, migration, and lymph node metastasis [37]. Nevertheless, the tumor-suppressive effects of FTO on gliomas have been reported. Tao *et al*. [29] reported that FTO protein contents were downregulated in gliomas compared with normal tissue samples. Consistently, we also observed downregulated mRNA expression and decreased protein levels of FTO in glioma intermediate and core regions, as well as hypoxia-challenged glioma cells. The aberrant expression levels of FTO suggest its potential role in glioma adaptation to hypoxia.

Although FTO has been recognized as an oncogene in several cancers, research indicating FTO as a tumor-suppressive gene also demonstrated that the expression level of FTO was remarkably downregulated within several tumors. FTO exerts a protective effect on hepatocellular carcinoma (HCC) [38]. FTO downregulation was linked to tumor size, metastasis, and vascular invasion, which predicted impaired prognosis [39]. FTO expression was decreased in clear cell renal cell carcinoma (ccRCC) tissue samples. FTO partially promoted the anti-tumor properties of ccRCC by decreasing m6A levels of PPARγ coactivator (PGC)‐1α mRNA transcripts and therefore increasing PGC-1α expression [40]. FTO hindered ovarian cancer stem cell self-renewal by m6A RNA demethylation and inhibited tumor development by suppressing cAMP signaling [41]. Regarding glioma, FTO interacts with FOXO3a to promote its transcriptional activity and suppresses glioma aggressiveness [29]. In this study, we demonstrated that FTO overexpression inhibited glioma cell aggressiveness under hypoxic conditions by inhibiting cell proliferation, migration, and invasion; in addition, m6A RNA methylation levels were also decreased in glioma cells. *In vivo*, FTO overexpression also inhibited tumor growth in a xenograft model. However, FTO expression is downregulated in glioma, particularly in the core and intermediate regions; therefore it is necessary to investigate the contributor(s) to FTO downregulation.

miR-27a-3p is a well-known oncogenic miRNA in cervical cancer [42], breast cancer [43], renal cell carcinoma [44], gastric cancer [45], and bladder cancer [46]. Regarding glioma, miR-24-3p and miR-27a-3p enhance glioma cell proliferative ability through cooperative regulation of MXI1 [47]. In this study, in contrast to FTO, miR-27a-3p expression was upregulated in the intermediate and core regions of gliomas, as well as in hypoxia-challenged glioma cells. Online prediction tools and experimental analyses indicated the binding of miR-27a-3p to FTO. miR-27a-3p directly binds to FTO to dramatically inhibit its expression in hypoxia-challenged glioma cells. Consistent with previous studies on the effects of miR-27a-3p on cancers, miR-27a-3p serves as an oncogenic miRNA in glioma by enhancing the malignant behaviors of glioma cells under hypoxia. Furthermore, FTO overexpression partially abolished the effects of miR-27a-3p on hypoxia-challenged glioma cells, suggesting that FTO is downstream of miR-27a-3p, eliminating the oncogenic effects of miR-27a-3p.

The transcription factors of the forkhead box O (FOXO) family regulate a wide range of gene expression programs and influence a wide range of cellular activities, including cell cycle regulation, cell survival, and metabolism [48]. FOXO3a, a member of the FOXO family of transcription factors, regulates the expression of its target genes to influence essential cellular processes such as programmed cell death and proliferation [49]. The FOXO3a/BIM axis has been reported to affect the chemosensitivity of BMP4-differentiated glioma stem cells to temozolomide [50]. In BV173 cells, FoxO3a may transactivate the BCL6 promoter; active FoxO3a is linked to up-regulation of BCL6 and down-regulation of cyclin D2 expression [51]. Furthermore, BNIP3 [52] and PUMA [53] have been reported as direct downstream targets of miR-27a. More importantly, FTO has been reported to serve as a tumor suppressor in gliomas by interacting with FOXO3a, enhancing FOXO3a nuclear translocation and target gene expression [29]. Considering these previous findings, FTO might exert its role in glioma by interacting with FOXO3a and modulating FOXO3a target gene expression. Consistent with this speculation, FTO overexpression dramatically increased, whereas miR-27a-3p overexpression decreased the levels of nuclear FOXO3a, as well as the protein levels of FOXO3a downstream BIM, BNIP3, BCL-6, and PUMA. Moreover, FTO overexpression partially eliminated the miR-27a-3p-mediated negative regulation of FOXO3a nuclear translocation and downstream target expression. These findings suggest that FTO might interact with FOXO3a, promoting FOXO3a nuclear translocation and enhancing FOXO3a target expression.

Conclusively, FTO serves as a tumor suppressor in glioma by suppressing hypoxia-induced malignant behaviors of glioma cells. miR-27a-3p is a major contributor to FTO downregulation in glioma under hypoxia.

Nevertheless, although the protein levels of HNRNPC, METTL14, METTL3, FTO, IGF2BP3, ALKBH5, and IGF2BP2 were determined in target tissues and cell lines, only the specific role of FTO was investigated in this study. In our future study, the specific roles of these factors in hypoxia-related mechanisms in glioma tumorigenesis will be investigated, and the understanding of m6A RNA methylation functions in glioma development should be improved.

## Supporting information

019supplementary_data

## Supplementary Data

Supplementary data is available at *Acta Biochimica et Biophysica Sinica* online.
