# Supplementary material for: The miR-27a-3p/FTO axis modifies hypoxia-induced malignant behaviors of glioma cells: miR-27a-3p/FTO affects glioma growth
Source: Acta Biochim Biophys Sin (Shanghai). 2023 Jan 31;55(1):103–16. doi: 10.3724/abbs.2023002 (PMC10157519; doi:10.3724/abbs.2023002)
Supplement: 019supplementary_data [file 019supplementary_data.pdf]

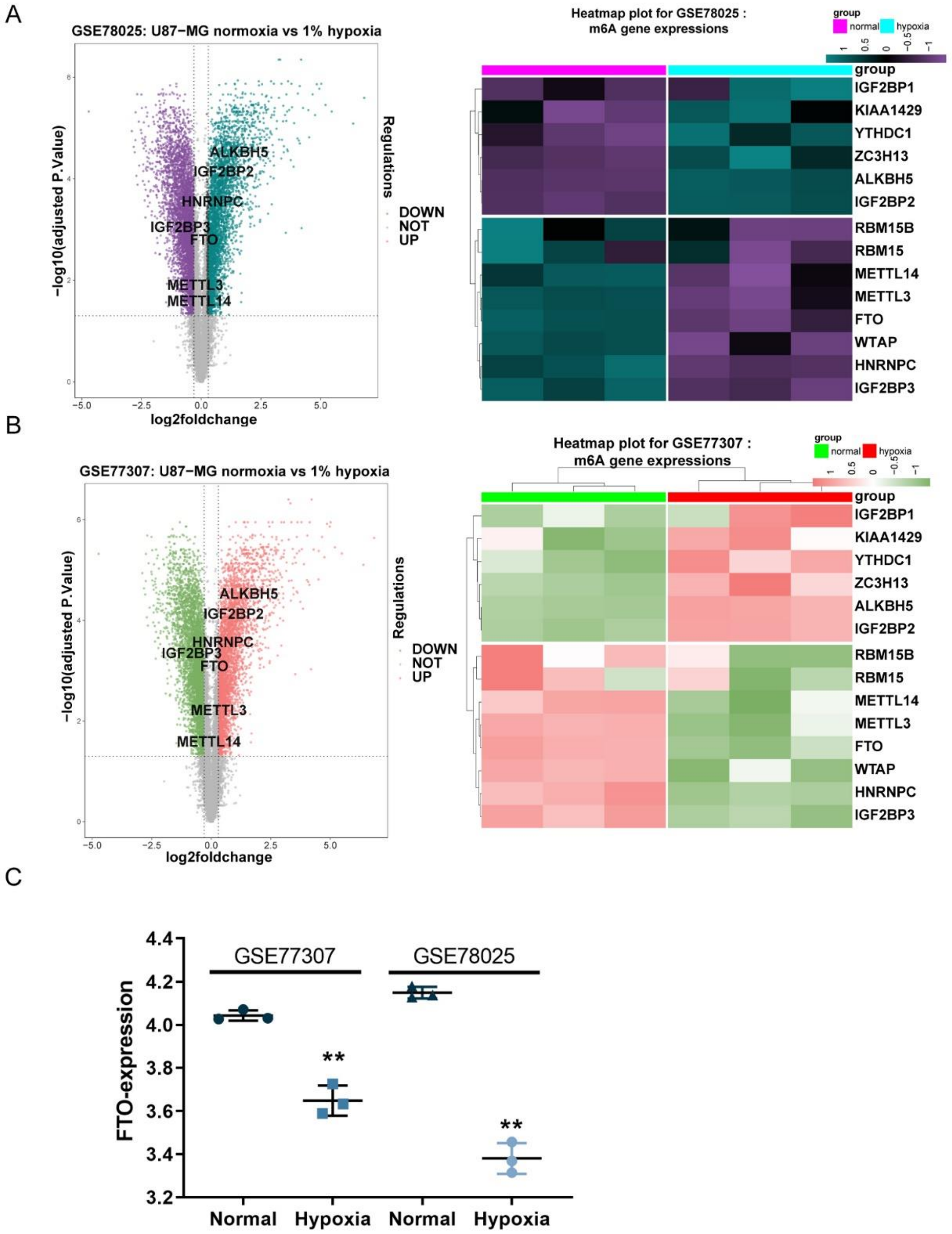

Supplementary Figure S1. FTO expression is decreased in hypoxia-treated glioma cells Differentially expressed m6A RNA methylation regulators in glioma cells under normoxia or hypoxia (1% O_2) according to GSE78025 (A) and GSE77307 (B). (C) The expression level of FTO in those two GSE datasets.

Supplementary Table S1. The sequence of shRNA, miRNAs and vector construction

	Forward (5′→3′)	Reverse (5′→3′)
**RT-qPCR**		
FTO	AACACCAGGCTCTTTACGGTC	TGTCCGTTGTAGGATGAACCC
Tubulin	ACCAACCTACGGGGATCTGAA	TTGACTGCCAACTTGCGGA
PCNA	CCTGCTGGGATATTAGCTCCA	CAGCGGTAGGTGTCGAAGC
Vimentin	AGTCCACTGAGTACCGGAGAC	CATTTCACGCATCTGGCGTTC
Twist	GTCCGCAGTCTTACGAGGAG	GCTTGAGGGTCTGAATCTTGCT
miR-27a-3p	RT : GTCGTATCCAGTGCGTGTCGTGG AGTCGGCAATTGCACTGGATACG ACGCGGAA	
	F: GCCGTTCACAGTGGCTAAG	R: CAGTGCGTGTCGTGGAGT
LeT-7i-5p	RT : GTCGTATCCAGTGCGTGTCGTGG AGTCGGCAATTGCACTGGATACG ACAACAGC	
	F: GCCGGTGAGGTAGTAGTTTGT	R: CAGTGCGTGTCGTGGAGT
miR-382-3p	RT : GTCGTATCCAGTGCGTGTCGTGG AGTCGGCAATTGCACTGGATACG ACAAGTGT	
	F: GCCAATCATTCACGGACA	R: CAGTGCGTGTCGTGGAGT
miR-23b-3p	RT : GTCGTATCCAGTGCGTGTCGTGG AGTCGGCAATTGCACTGGATACG ACGGTAAT	
	F: GCATCACATTGCCAGGG	R: CAGTGCGTGTCGTGGAGT
miR-140-5p	RT : GTCGTATCCAGTGCGTGTCGTGG AGTCGGCAATTGCACTGGATACG ACCTACCA	
	F: GCGCAGTGGTTTTACCCTA	R: CAGTGCGTGTCGTGGAGT
U6	F: CTCGCTTCGGCAGCACA	R: AACGCTTCACGAATTTGCGT
**Transfection**		
Lv-FTO	CTACCGGACTCAGATCTCGAGAT GAAGCGCACCCCGACT	GTACCGTCGACTGCAGAATTCCTAGGGTT TTGCTTCCAGAAGC
lv-sh-NC	GATCCGCAGATGAAGGCACGGT CACGCTCGAGGCAGATGAAGGC ACGGTCACGTTTTTG	AATTCAAAAAGCAGATGAAGGCACGGTC ACGCTCGAGGCAGATGAAGGCACGGTCA CG
lv-sh1-FTO	GATCCGTTCAAGAAGCCTTTCTC ACACTCGAGTGTGAGAAAGGCTT CTTGAACTTTTTG	AATTCAAAAAGTTCAAGAAGCCTTTCTCA CACTCGAGTGTGAGAAAGGCTTCTTGAAC G

lv-sh2-FTO	GATCCGCTATTTCATGCTTGATGATCCTCGAGGATCATCAAGCATGAAATAGCTTTTTG	AATTCAAAAAGCTATTTCATGCTTGATGATCCTCGAGGATCATCAAGCATGAAATAGCG
miR-27a-3p **aGomir**	UUCACAGUGGCUAAGUUCCGC	GGAACUUAGCCACUGUGAAUU
miR-27a-3p antagomir	GCGGAACUUAGCCACUGUGAA	
Wt-FTO	AATTCTAGGCGATCGCTCGAGATCATGTGTTTGAGGATTTCATGTG	ATTTTATTGCGGCCAGCGGCCGCATCACTTTTCTCAAGAGACCTGGG
Mut-FTO	TTCTGCCACAGGTTGGAGAAGGACTTTCCTGGAA	TCCAACCTGTGGCAGAAGCAAGCCTTCCTTTGAC
